# Tweets by People With Arthritis During the COVID-19 Pandemic: Content and Sentiment Analysis

**DOI:** 10.2196/24550

**Published:** 2020-12-03

**Authors:** Danielle Berkovic, Ilana N Ackerman, Andrew M Briggs, Darshini Ayton

**Affiliations:** 1 School of Public Health and Preventive Medicine Monash University Melbourne Australia; 2 School of Physiotherapy and Exercise Science Curtin University Perth Australia

**Keywords:** COVID-19, SARS-CoV-2, novel coronavirus, social media, Twitter, content analysis, sentiment analysis, microblogging, arthritis

## Abstract

**Background:**

Emerging evidence suggests that people with arthritis are reporting increased physical pain and psychological distress during the COVID-19 pandemic. At the same time, Twitter’s daily usage has surged by 23% throughout the pandemic period, presenting a unique opportunity to assess the content and sentiment of tweets. Individuals with arthritis use Twitter to communicate with peers, and to receive up-to-date information from health professionals and services about novel therapies and management techniques.

**Objective:**

The aim of this research was to identify proxy topics of importance for individuals with arthritis during the COVID-19 pandemic, and to explore the emotional context of tweets by people with arthritis during the early phase of the pandemic.

**Methods:**

From March 20 to April 20, 2020, publicly available tweets posted in English and with hashtag combinations related to arthritis and COVID-19 were extracted retrospectively from Twitter. Content analysis was used to identify common themes within tweets, and sentiment analysis was used to examine positive and negative emotions in themes to understand the COVID-19 experiences of people with arthritis.

**Results:**

In total, 149 tweets were analyzed. The majority of tweeters were female and were from the United States. Tweeters reported a range of arthritis conditions, including rheumatoid arthritis, systemic lupus erythematosus, and psoriatic arthritis. Seven themes were identified: health care experiences, personal stories, links to relevant blogs, discussion of arthritis-related symptoms, advice sharing, messages of positivity, and stay-at-home messaging. Sentiment analysis demonstrated marked anxiety around medication shortages, increased physical symptom burden, and strong desire for trustworthy information and emotional connection.

**Conclusions:**

Tweets by people with arthritis highlight the multitude of concurrent concerns during the COVID-19 pandemic. Understanding these concerns, which include heightened physical and psychological symptoms in the context of treatment misinformation, may assist clinicians to provide person-centered care during this time of great health uncertainty.

## Introduction

Social media contains a plethora of health information pertaining to individuals living with chronic illness [1,2]. Social media provides a unique opportunity to observe thoughts, feelings, and interactions between individuals living with chronic illness, and to leverage this information to promote positive health outcomes [3,4]. The COVID-19 pandemic has seen widespread uptake of social media use. Twitter, a well-known social media platform primarily used for microblogging, plays a significant role in crisis communications and can be a powerful tool to communicate to entire populations during a time of rapid change [5]. Twitter is already frequently used by individuals with arthritis to communicate with peers [6] and to receive up-to-date information from health professionals and services about novel therapies and management techniques [7].

Many inflammatory arthritis medications act as immunosuppressants, which are advantageous in controlling arthritis-mediated inflammatory responses, but can increase the risk of infection [8]. Long-term use of immune-modulating therapies or glucocorticoids may place individuals with arthritis in a higher-risk category for contracting the novel coronavirus SARS-CoV-2, although the level of risk is poorly understood [9-12]. Current guidelines suggest that individuals living with arthritis should physically distance from other individuals and their communities [13], and will likely need to do so for a longer duration than the general public. Heightened stress due to potential medication shortages, reduced opportunities to personally consult health care professionals, and enforced limitations on physical activity (which, for many, is a core component of arthritis self-management [14]) contribute to worsening arthritis symptoms, including disease flares. At present, individuals with arthritis have already reported increased physical pain and psychological distress during the COVID-19 pandemic [15,16].

In the current COVID-19 outbreak, Twitter’s overall daily usage has surged by 23% in 2020 [17], presenting a unique opportunity to assess the content and sentiment of tweets. Examining publicly available tweets allows exploration of important proxy topics through microblogging data, without directly burdening this population. This research aims to identify proxy topics of importance for people with arthritis (of any diagnostic category) during the COVID-19 pandemic by characterizing the textual content and sentiment of tweets, and to explore the emotional context of tweets by people with arthritis during the early phase of the pandemic.

## Methods

### Design

An exploratory content and sentiment analysis was undertaken. All data were collected and reported according to the terms and conditions of Twitter, which state that content posted by individuals is publicly available to syndicate, broadcast, distribute, retweet, promote, or publish, excluding private information (eg, home addresses or identity documents) [18]. Use of tweets by individuals outside of Twitter can be carried out with no compensation paid to the individual tweeter, as use of Twitter is agreed upon as sufficient compensation [18]. The Monash University Human Research Ethics Committee (Project ID 24354) approved this project.

### Inclusion and Exclusion Criteria

Publicly available tweets posted in English or with English translation (automated through Twitter), with the hashtags shown in Table 1 were included.

**Table 1:** Hashtags categorized by topic.

Topic	Hashtag
COVID-19	#coronavirus #covid19 #isolation #socialdistancing
Arthritis	#arthritis #spoonie #rheumatologist #rheumatology

**Figure 1:**
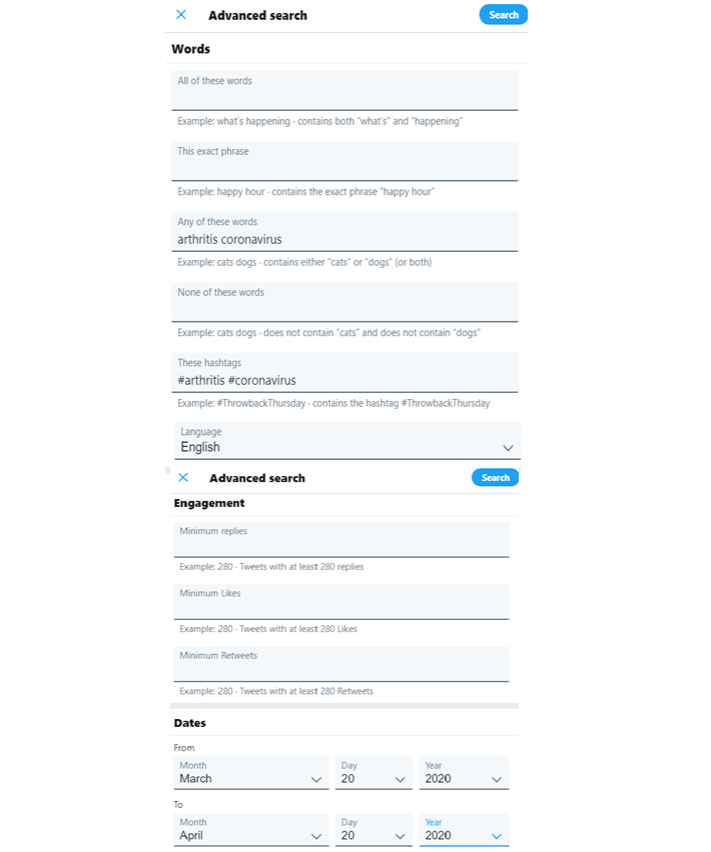
The advanced search function on Twitter [19].

Hashtags were selected based on trialing various combinations through Twitter’s search function. The highest number of tweets retrieved were for the hashtags #coronavirus, #covid19, #arthritis, and #spoonie (the latter term coined by people living with chronic illness to describe various methods of pain management [7]). Hashtags were also searched as words; for example, where #arthritis was searched, arthritis without a hashtag was searched as well. This ensured that specific arthritis types mentioned in tweets without hashtags (eg, rheumatoid arthritis or psoriatic arthritis) were included. Hashtags and words were combined through the advanced search function on Twitter using the domains Hashtags, Words, Language, and Dates (Figure 1 [19]). Tweets were excluded if they originated from organizations, news outlets, or health professionals rather than individuals in order to focus on the personal perspective.

### Data Collection

Tweets were retrospectively extracted from March 20 to April 20, 2020. The search strategy and search results are included in Multimedia Appendix 1. The search timeframe was chosen to align with the early phase of the COVID-19 pandemic and the period when many developed countries (eg, the United States, the United Kingdom, Italy, Australia) announced enforceable physical distancing or isolation measures [20-23]. The desktop version of the Twitter website (versus the mobile app) was used for data collection for ethical purposes with only publicly available tweets extracted, rather than through a private login. In addition to the tweets themselves, accompanying data fields were extracted from each tweet using a customized template. Extracted data fields included (where possible): Twitter profile blurb, gender of tweeter, country of tweeter, number of likes, number of retweets, number of replies, hashtags used, number of hashtags, and use of accompanying photos. Data were stored in a Microsoft Excel spreadsheet (v16.0, Microsoft Corp).

### Data Analysis

To address the research aims, two data analysis techniques were utilized: summative content analysis and sentiment analysis. Content analysis was used to characterize the textual contents of tweets related to arthritis and COVID-19. Content analysis is exploratory; it aims to quantify and describe unknown phenomena [24]. During the content analysis process, the primary researcher (DB) read each tweet and categorized the tweets into a representative theme and subtheme related to a topic of importance for people with arthritis during the study period.

Summative content analysis occurred through a process of coding, which involved counting and comparisons of Twitter content, followed by interpretation of the underlying context [25]. To begin with, the first 10 tweets were analyzed and allocated a summary code. The code represents the theme of a tweet (eg, “health care experiences”). As additional tweets were examined, they were given one of the original codes or allocated a new code based on new content. This process was repeated until each tweet was coded and themed. Once these original themes were finalized, they were recoded for additional context, and a second researcher (DA) checked the coding. For example, “health care experiences” was coded for similarities in people’s health care experiences, such as difficulties accessing medications. Given Twitter’s character limits, each tweet only contained one theme. The frequency of original themes and subthemes was counted to indicate importance [26].

Sentiment analysis enables an examination of written and spoken words for positive and/or negative emotion. When applied to health care or social media research, sentiment analysis facilitates interpretation of textual information about patient experience from a person-centered perspective [27]. Once tweets were coded and categorized into themes, sentiment analysis was employed to assess the emotion associated with the theme using Glaser and Strauss’s [28] 6 codes for sentiment analysis, a common framework used for Twitter-based research [29,30]:

1. No sentiment: the tweet has no emotion or words or special punctuation; is matter-of-fact sounding;
2. Wretched: the tweet is purely negative;
3. Bad: the tweet contains mainly negative phrases and words that outweigh any positive sentiment;
4. So-so: the tweet has a mediocre and balanced sentiment where positive and negative statements are balanced;
5. Swell: the tweet contains mainly positive phrases and words which outweigh negative sentiment;
6. Great: the tweet is purely positive.

The presence of emojis, which are shorthand facial expression symbols that are frequently used to facilitate communication of mood and emotion, in tweets were also analyzed. To provide information regarding the emotional content of the tweets, Emoji Sentiment Ranking, as outlined by Kralj Novak et al [31], was applied. Tweets containing an emoji were categorized into one of three sentiment scores: (1) negative, (2) neutral, and (3) positive. Together, the content and sentiment analyses provide a proxy indicator of the topics of interest for, and perceived emotions of, people with arthritis during the COVID-19 pandemic.

Even in social media studies, it is imperative to protect participant anonymity [32]. To avoid reverse identification of participants based on their tweets (which can be found through internet searches), tweets analyzed in this study are not quoted verbatim. Instead, all data are expressed in aggregate form through descriptive statistics and qualitative syntheses.

## Results

The analysis included 149 tweets posted during the study period. The majority of tweeters were female and based in the United States. The most common arthritis type was rheumatoid arthritis. Table 2 outlines gender, country of residence, and arthritis type.

**Table 2:** Demographics of Twitter users sampled.

Characteristic		Users (N=149), n (%)
**Gender**		
	Female	105 (70.0)
	Male	31 (21.0)
	Unknown	13 (9.0)
**Country**		
	United States	68 (45.5)
	United Kingdom and Northern Ireland	39 (26)
	Canada	18 (12.0)
	Australia	1 (0.7)
	France	1 (0.7)
	Germany	1 (0.7)
	India	1 (0.7)
	New Zealand	1 (0.7)
	Unknown	19 (13.0)
**Diagnostic category**		
	Arthritis (specific arthritis type unclear)	86 (58.0)
	Rheumatoid arthritis	32 (21.0)
	Systemic lupus erythematosus	12 (8.0)
	Psoriatic arthritis	10 (7.0)
	Ankylosing spondylitis	3 (2.0)
	Osteoarthritis	3 (2.0)
	Juvenile idiopathic arthritis	3 (2.0)

Content analysis revealed seven themes from the tweets: (1) health care experiences, (2) personal stories, (3) links to or advertisements of relevant blogs, (4) discussion of arthritis-related symptoms, (5) advice sharing, (6) messages of positivity, and (7) stay-at-home messaging. Table 3 details the original themes and subthemes.

**Table 3:** Content analysis of themes and subthemes.

Theme and subthemes		Tweets, n (%)	Examples of phrases or #hashtags describing content
**Health care experiences**		55 (37.0)	“I’m a long-term user of #hydroxychloroquine” “#Hydroxychloroquine destroyed my red blood cells”
	Difficulties accessing hydroxychloroquine	20 (36.5)	
	Past experiences using hydroxychloroquine	20 (36.5)	
	Support for President Trump’s advice to use hydroxychloroquine to cure COVID-19	9 (16.0)	
	Experiences within the National Health Service (UK NHS)	4 (7.0)	
	Managing medication changes during COVID-19	2 (4.0)	
**Personal stories**		29 (20.0)	“My rheumatologist has asked that I go into isolation. Now all I can do is enjoy the world from my window” “My immune system is compromised but I’m being told to go to a germy hospital???”
	Explanation of history of managing arthritis, and subsequent fears of contracting or dying from COVID-19	9 (31.0)	
	Description of ways to self-manage physical symptoms (eg, exercising, staying connected with friends)	7 (24.0)	
	Physical and psychological challenges of socially distancing	7 (24.0)	
	Perceived barriers to attending rheumatologist appointments (eg, discomfort of wearing masks, fear of entering a high-risk location)	6 (21.0)	
**Links to or advertisements of relevant blogs and forums**		22 (14.0)	“To our members, subscribers, followers, and fans: we are here for you. #BeSafe”
	Recommendations and links from individuals to official patient- and consumer-led blogs (eg, CreakyJoints)	10 (45.0)	
	Personal blogs on individual COVID-19 experiences (eg, how to manage worsening symptoms)	7 (32.0)	
	Unofficial patient blogs (eg, online communities and forums) to create support networks for individuals	5 (23.0)	
**Discussion of arthritis-related symptoms**		15 (10.0)	“Anyone else’s arthritis flaring due to extra phone use?”
	Increased physical pain	11 (73.5)	
	Difficulty sleeping	2 (13.5)	
	Reduced dexterity	2 (13.5)	
**Advice seeking and sharing**		14 (9.0)	“Any suggestions for chronic pain sufferers? Coronavirus has been very rough #arthritis #spoonie” “Do we know if those of us with autoimmune conditions (arthritis) have a higher risk from #coronavirus”
	Questions directed at government bodies (eg, the NHS and national working-from-home regulations)	5 (36.0)	
	Seeking advice from physiotherapists on at-home exercises to manage physical symptom burden	3 (22.0)	
	Advice on whether to temporarily cease taking immunosuppressant medications	2 (14.0)	
	Advice on how to protect airways if dexterity limitations prevent mask-wearing	2 (14.0)	
	Questions directed to delivery services regarding delays	2 (14.0)	
**Messages of positivity**		8 (6.0)	“It’s amazing how motivating isolation can be! On my bike but knee sore #arthritis”
	Gratitude for friends, family, and to still be able to appreciate life	4 (50.0)	
	Spare time as a result of physical distancing facilitating more time to exercise and reduce physical symptom burden	4 (50.0)	
**Stay-at-home messaging**		6 (4.0)	#stayathome #arthritissucks #arthritiswarrior
	Emotional appeals for people to stay at home	4 (67.0)	
	Angry appeals for people to stay at home	2 (33.0)	

The most common theme identified was experiences of navigating the health care system during the COVID-19 pandemic. Hydroxychloroquine (brand name Plaquenil) featured prominently in tweets, in terms of difficulties accessing the medication, past experiences using the medication, and recommendations from the President of the United States to use this medication to cure or prevent COVID-19, despite the lack of evidence or medical advice. Some individuals tweeted about their experiences within the National Health Service (NHS) in the United Kingdom, where patients were subject to longer-than-usual delays for rheumatology appointments, medication infusions, and general health check-ups.

Many individuals used Twitter as a platform to connect with and seek support from peers with arthritis. Tweets contained personal stories, links to personal and consumer-led blogs, discussion of arthritis-related symptoms, and advice seeking and sharing. Some individuals shared their challenges managing arthritis symptoms whilst being confined to their homes and questioned their physical and psychological capacity to function if they were to contract COVID-19. Others described the physical and emotional challenges associated with isolation, including increased physical pain, reduced dexterity, and missing family. Strategies to manage these symptoms included exercising and staying socially connected online with friends to ease mental strain. Tweeters were willing to guide others to potentially helpful resources, particularly blogs run by professional organizations (eg, CreakyJoints, the Arthritis Society [Canada]). Several tweets contained questions were directed toward government bodies (eg, regarding national working-from-home policies), whereas others reached out to physiotherapists or peers with arthritis for advice on appropriate exercises or lifestyle modifications to manage symptom burden during the isolation period. Some tweeters noted dexterity limitations that were highly relevant to COVID-19, such as being incapable of placing a mask behind their ears.

Some tweets were positive with tweeters noting they used their newfound spare time to concentrate on exercise, which was beneficial for mental health and pain reduction. Finally, some individuals expressed their desire for people to stay at home to flatten the curve of infections to return to normal life. Disapproval was voiced toward those refusing to practice physical distancing, whereas others expressed anger toward people not adhering to stay-at-home orders.

Sentiment analysis provided complementary information about the emotions associated with the content analysis themes. Table 4 details the original themes and corresponding sentiment, with phrases or hashtags describing tweeters’ personal experiences.

**Table 4:** Sentiment analysis of tweets.

Original theme and sentiment^a^		Tweets, n (%)	Examples of phrases or #hashtags
**Health care experiences**		55 (37.0)	
	Great	5 (9.0)	“doddle,” “virus gone in 3 days,” “NO SIDE EFFECTS,” “credible and good”
	Swell	3 (5.0)	“thankful,” “it must be working,” “don’t be afraid”
	So-so	8 (15.0)	“hope it helps,” “minimal risk,” “might work,” #BeWarnedBeWell, “it might fight COVID-19,” “take care world!” “interesting to see”
	Bad	6 (11.0)	#plaquenilshortage,” “no tests will be done for 2 months,” “facing shortages,” “no proof #Hydroxychloroquine makes us safe”
	Wretched	28 (51.0)	“seriously ill,” “madness,” “pissed off,” #coronavirushoax, #painsomnia, “f*ck all chance,” “made me sick,” “I could die,” “harmful results,” “side effects unbearable,” “I call bullsh*t,” “[medication] shortages & living with symptoms,” #EVIL!!, “shorten my life,” “life threatening for us,” “true danger,” “scared,” “fear a shortage,” #drugshortage, “will end up in hospital,” “write us all off,” “it made me so sick,” “NO real testing,” “it’s not safe”
	No sentiment	5 (9.0)	“research says,” “UK government wants,” “prove hypothesis,” “IL-6 is raised,” “from the CDC”
**Personal stories**		29 (20.0)	
	Great	5 (17.0)	“all good during this #covid19,” “it makes me feel better on a personal level,” “bring it on world,” “I am a champion!” #livingmybestlife
	Swell	5 (17.0)	#AloneTogether, #nevergiveup, #hope, #makethebestofit, “trying to keep active”
	So-so	3 (10.0)	“it is what it is,” “I hope they have a lot of masks and sanitizer!”
	Bad	2 (7.5)	“I didn’t want to risk heading out,” “limiting my usual walk”
	Wretched	12 (41.0)	“give us a break, FFS!” #HighRiskCovid19, “I hate #Coronavirus,” “literally a pain,” “holy sh*t,” “screaming into the void,” “I am not #expendable,” “being told to go to a germy hospital,” “my chances of surviving #covid19 are horrible,” “vulnerable patients like me”
	No sentiment	2 (7.5)	“info is changing daily,” “I’m immunocompromised because”
**Links to or advertisements of relevant blogs and forums**		22 (14.0)	
	Great	1 (5.0)	“so grateful”
	Swell	2 (9.0)	“talking about work/life balance,” “we are here for you”
	So-so	4 (18.0)	“coping in isolation,” “trying to deal,” “hoping to support others”
	Bad	2 (9.0)	“how to handle flares,” “learn what’s happening to people with arthritis”
	Wretched	4 (18.0)	“covid19 scariness,” “fear of dying,” “unpredictability and fear,” “we worry about everyone”
	No sentiment	9 (41.0)	“video games during covid19,” “questions I have,” “share with your networks,” “breaking news,” “please consider sharing,” “please retweet,” “I found this information”
**Discussion of arthritis-related symptoms**		15 (10.0)	
	Great	0 (0.0)	—^b^
	Swell	0 (0.0)	—
	So-so	1 (7.0)	“not the end of the world”
	Bad	1 (7.0)	“I have to take a break”
	Wretched	13 (86.0)	“struggling to sleep/be active,” “spoonie fail,” “the arthritis flared out of control,” “my shoulder is a casualty,” “pain in the knees,” “I’m screwed,” “ouchy grouchy,” “feet are burning,” “f*ck you coronavirus,” “worsening arthritis pain,” “I’m already achy,” “flaring due to extra phone use”
	No sentiment	0 (0.0)	—
**Advice seeking**		14 (9.0)	
	Great	0 (0.0)	—
	Swell	1 (7.0)	“thanks to the doctors for their expertise”
	So-so	0 (0.0)	—
	Bad	1 (7.0)	“some chance of developing complications”
	Wretched	8 (57)	“lowered immunity more than it already is,” “significantly increase risk of infection,” “things have been rough,” “zero immune system,” “so stressed about #coronavirus,” “no money for food let alone masks,” “I’m disabled”
	No sentiment	4 (29.0)	“grateful for info,” “where can I volunteer to get tested,” #lockdownUK, “would appreciate a video of exercises for in #lockdown”
**Messages of positivity**		8 (6.0)	
	Great	2 (25.0)	“coronavirus caused some good things to happen,” “amazing how motivating boredom is”
	Swell	3 (37.5)	#selfmanagement, “right exercises to keep arthritis at bay,” “enjoy the little things”
	So-so	1 (12.5)	“life is too short to be scared”
	Bad	1 (12.5)	“keeping positive a challenge”
	Wretched	0 (0.0)	—
	No sentiment	1 (12.5)	“this information might help”
**Stay-at home-messaging**		6 (4.0)	
	Great	0 (0.0)	—
	Swell	0 (0.0)	—
	So-so	0 (0.0)	—
	Bad	0 (0.0)	—
	Wretched	5 (83.0)	“#selfisolation only because of my immune system,” #arthritissucks so stay the f*ck home,” “coronavirus could kill me #StayHomeSaveLives,” “#PLEASESTAYHOME I’m devastated,” “arthritis shot to sh*t stay home”
	No sentiment	1 (17.0)	#GoHomeStayHome

^a^Great: the tweet is purely positive; swell: the tweet contains mainly positive phrases and words that outweigh negative sentiment; so-so: the tweet has a mediocre and balanced sentiment where positive and negative statements are balanced; bad: the tweet contains mainly negative phrases and words that outweigh any positive sentiment; wretched: the tweet is purely negative; no sentiment: the tweet has no emotion or words or special punctuation and is matter-of-fact sounding.

^b^Not available.

A few tweets contained messages of positivity. While overall “keeping positive [was] a challenge,” some people encouraged others to “enjoy the little things” and that “life is too short to be scared.” Still, the extent to which people with arthritis were concerned for their health was evident in people’s stay-at-home messaging. Tweeters were notably anxious and angry, writing that “#arthritissucks so stay the f*ck home,” that “coronavirus could kill me #StayHomeSaveLives,” and “#PLEASESTAYHOME I’m devastated.”

Using Glaser and Strauss’s [28] classifications, more than half of the tweets contained wretched (purely negative) or bad (mainly negative) sentiment (n=83, 56%), whereas only one-fifth of tweets contained great (purely positive) or swell (mainly positive) sentiment (n=27, 18%). In total, 16 (11%) tweets contained sentiment that was so-so (balanced negativity and positivity), and 22 (15%) tweets contained no sentiment (matter-of-fact sounding).

Individuals in the United States appeared particularly despondent regarding their health care experiences during the COVID-19 pandemic. When referencing interactions with the health care system, tweets contained phrases such as “true danger,” “seriously pissed off,” and “scared.” Tweeters noted “it’s not safe” for people with arthritis facing hydroxychloroquine shortages, and that the #drugshortage was “life threatening.” Some individuals mused that there is “minimal risk” trying hydroxychloroquine to cure COVID-19, and that it might be “interesting to see” the results of this medication. Outside of the United States, Canadians tweeted that engaging with the health care system during the pandemic was a “doddle” and that they were “thankful” to continue to have access to their health professionals.

Many individuals described their personal stories negatively. People with arthritis discussed barriers to accessing care, such as being surprised at “being told to go to a germy hospital” and that they “didn’t want to risk going out.” Tweets with links to blogs were accompanied by captions “fear of dying,” “unpredictability and fear,” and “we worry about everyone.” Some were more positive, encouraging followers that they are “hoping to support others.” Although their representation was small, people with arthritis in Ireland and New Zealand viewed the isolation measures as an opportunity for #hope, encouraging others to #makethebestofit and to #nevergiveup.

A consistent sentiment was that people with arthritis were negative in their discussion of physical and psychological symptoms, with many individuals seeking advice from peers and health professionals to remedy symptoms. Tweets highlighted the spectrum of symptoms that individuals experienced during the pandemic, with people “struggling to sleep/be active,” experiencing “worsening arthritis pain,” and having “pain in the knees.” Some noted that attempting to manage physical symptoms while in isolation was “literally a pain” and that the psychological toll was like “screaming into the void.” One tweeter mentioned that their arthritis was “flaring due to extra phone use,” which was frustrating since this was a primary method of maintaining social connection and communication with family or colleagues during the pandemic. Similarly, individuals tweeting and asking for advice did so by prefacing that “things have been rough,” that they have “zero immune system,” and that they are “so stressed about #coronavirus.”

Our sentiment analysis included an overview of emoji use in tweets, as summarized in Table 5.

**Table 5:** Sentiment analysis of emoji use in tweets.

Emoji^a^	Count, n	Emoji name	Phrases or #hashtags accompanying the emoji	Emotion	Link to original themes
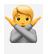	3	Face with no good gesture	“my feet are burning,” things are very painful,” “my chances of surviving are horrible”	Negative	Discussion of arthritis-related symptoms, personal stories
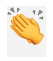	2	Clapping hands sign	“my team have worked so hard,” “massive thanks”	Positive	Links to or advertisements of relevant blogs and forums, advice seeking
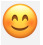	2	Smiling face with smiley eyes	“hope it helps all”	Positive	Health care experiences
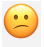	2	Confused face	“I might be screwed,” “I think my consultant is wrong”	Negative	Health care experiences, discussion of arthritis-related symptoms
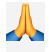	2	Person with folded hands	#lupuswarrior	Positive	Personal stories, health care experiences
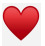	2	Red heart suit	“hoping to support others,” #BeSafe	Positive	Links to or advertisements of relevant blogs and forums
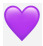	2	Purple heart	“choose to spread awareness”	Positive	Personal stories
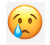	1	Crying face	“#coronavirus has opened my eyes”	Neutral	Health care experiences
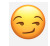	1	Smirking face	“#makingthebestofit	Positive	Personal stories
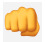	1	Fisted hand sign	“I’ve got a morphine patch on to help”	Positive	Personal stories
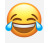	1	Face with tears of joy	“happy sitting in the sun”	Positive	Messages of positivity
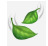	1	Leaves fluttering in the wind	“I’m laying outside”	Positive	Personal stories
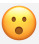	1	Face with mouth open	“could potentially lead to the cure for #Coronavirus”	Positive	Health care experiences
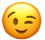	1	Winking face	“I made healthy Easter treats in self islation”	Positive	Personal stories
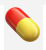	1	Pill	“I really miss anti-inflammatories”	Negative	Stay-at-home messaging
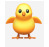	1	Front-facing baby chick	#livingmybestlife	Positive	Personal stories
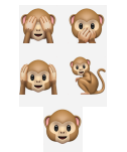	1 each	See-no-evil monkey, speak-no-evil monkey, hear-no-evil monkey, monkey, monkey face	“my rheumatologist said I wasn’t at high risk”	Positive	Health care experiences

^a^Emoji Sentiment Ranking did not include the upside-down face, the spectacle face, or the high-five emoji. There was one of each included in our data collection.

The emoji within tweets established tone and emphasized emotions. Most tweets used an emoji to express positive sentiment, such as clapping hands to thank colleagues for their support and a person with folded hands in acknowledgment of the personal commitment to the ongoing management of arthritis symptoms in isolation. The main emoji used to express negative sentiment were the face with “no good” gesture, and the confused face. Tweeters used the face with “no good” gesture to highlight their physical pain, and the confused face was used to express confusion around what arthritis patients perceived to be ill-informed health advice during the pandemic.

## Discussion

### Principal Findings

This study aimed to identify proxy topics of importance for people with arthritis during COVID-19 by characterizing the textual content and sentiment of tweets, and exploring the emotional context of tweets by people with arthritis during the early phase of the pandemic. We anticipated that this novel approach would ascertain contemporary topics of importance for immunocompromised and isolated individuals. Content analysis revealed seven themes relating to health care experiences, and sentiment analysis revealed that the majority of tweets contained negative emotion, particularly around medication shortages, increased arthritis symptoms, and the physical and mental toll of physical distancing or living in isolation. Our findings provide a starting point for understanding the impacts of COVID-19 on vulnerable arthritis populations and provide some insights for physicians and researchers regarding current concerns that may inform tailored care.

More than one-third of tweets discussed health care experiences, primarily focusing on the reduced availability of hydroxychloroquine. Individuals were highly anxious about hydroxychloroquine shortages after preliminary research found that the drug might act as a potential preventive measure, or possible cure, for COVID-19 and these early findings were highly publicized around the world. The original published article has since been retracted after researchers were unable to verify the reliability of their results [33]. Regardless, President Trump tweeted his support for hydroxychloroquine as “one of the biggest game changers in the history of medicine” [34], causing panic about medication shortages, particularly from those with systemic lupus erythematosus for which it is the first-line therapy [35]. Due to off-label prescriptions and hoarding practices, difficulties accessing hydroxychloroquine have been reported globally [36]. Hydroxychloroquine shortages pose a threat to the health and safety of people with inflammatory arthritis, with reports that many will experience flare-ups and may develop irreversible organ damage without their regular dose [35]. Our findings highlight the need for accurate information about treatments and their effectiveness and the critical role that clinicians play in dispelling myths and inaccuracies during times of rapidly changing information. A growing body of literature describes the potential benefits of using Twitter in clinical settings, and reports ways that clinicians are using platforms such as Twitter to communicate health information to the broader population [37-39]. In the context of infectious diseases in particular, evidence suggests that Twitter is beneficial to translate real-time clinical information [40,41]. Use of Twitter, being a component of mobile health (mHealth), also empowers patients to more positively perceive their abilities to manage chronic illness [42]. This presents an opportunity for clinicians and professional societies to use social media platforms such as Twitter to overcome evidence dissemination methods (eg, peer-reviewed articles and care guidelines) that are traditionally slow. Together, clinicians and patients can contribute to care in adults with arthritis, encouraging positive health outcomes throughout, and beyond, the pandemic.

Several tweets contained very personal narratives that highlighted individual fears of contracting COVID-19, and the challenges associated with being vulnerable to infection. Individuals described perceived barriers to accessing care, citing discomfort caused by wearing medical-grade personal protective equipment. Tweets contained accounts of neck pain attributed to wearing a mask; a documented side effect in other vulnerable populations [43]. Fitting a mask is also a dexterous task that some people with arthritis struggled to perform, and as a result, encountered abuse from others when buying groceries or going for a walk. The long-term psychosocial impact of this stigma should receive consideration in future research. Clinicians should consider COVID-19–related functional concerns for physically impaired patients, and incorporate new aspects of information-seeking into clinical consultations. For example, asking patients about their degree of difficulty with COVID-19–related functional tasks would help to elicit relevant functional challenges faced by people with arthritis and inform the provision of tailored patient education. Asking these questions also contributes to factors beyond patient education and care, such as facilitating access to nonacute symptom or pain management services such as allied health. These health services still need to be maintained during and after the pandemic, and for musculoskeletal health in particular, there is emerging commentary around the physical and psychosocial impacts of inhibited access [44,45].

Tweets detailed marked increases in general physical symptom burden; a concerning prospect as COVID-19 has impacted face to face consultations, with indications that disruptions to traditional service models will likely persist for some time. Evidence about the utility of telemedicine to manage pain is emerging, with consideration of barriers to implementation, and potential inequity in access [46], although health systems have been generally slow to implement this approach at scale. While the included tweets provide preliminary information about the growing symptom burden during the pandemic, the collection of systematic patient-reported outcomes data is needed to ensure that health care services are meeting the needs of people with arthritis during and after the COVID-19 pandemic.

A proportion of tweets related to social connection, that is, people reaching out to likeminded peers with arthritis through potentially informative blogs. Most links provided were to official (eg, CreakyJoints) or unofficial (eg, online communities and forums) blogs and provided information on how to manage physical and mental health in isolation, a range of arthritis-appropriate exercises, and existing evidence on the association between arthritis and risk of COVID-19 infection. Access to resources that are relevant, credible, and trustworthy appears to have been challenging for people with arthritis throughout the pandemic [47], and combined with high levels of misinformation online [48,49], this may account for the recent growth of platforms such as Twitter for sourcing information and advice.

Before the COVID-19 pandemic, people with arthritis primarily used social media for self-expression and positive messaging [50,51]. Our sentiment analysis (enhanced by classifying emojis to further characterize common emotions) demonstrates that the role of Twitter has evolved throughout the current pandemic to act as a space for people to share symptoms; to reach out to peers, organizations, and health professionals for information; and to create a sympathetic community of care. This is advantageous as it fosters connection between individuals with shared experiences but conversely may enable proliferation of misinformation [52]. Already, Twitter has been shown to inform clinical practice by capturing the experiences of patients with multiple sclerosis during the pandemic [53]. Understanding the COVID-19–related concerns of people with arthritis is also key to providing person-centered care and reducing distress during these rapidly changing times.

### Strengths and Limitations

The observational exploratory nature of this study enabled us to examine topics of importance for individuals with arthritis through a person-centered lens, without ethical issues or compromising the well-being of immunocompromised patients during the pandemic. Social media research is still in its infancy, and this novel method of data collection demonstrates the concerns of people with arthritis during a time of peak anxiety. There is some indication that tweeters were representative of the general inflammatory arthritis population; the majority were female and the most common arthritis type identified was rheumatoid arthritis. Nearly half (44.0%) of tweeters were based in the United States, which currently leads the world in COVID-19 cases and deaths [54].

We also acknowledge the research limitations. It is important to note that only 15% of adults regularly use Twitter, and that younger adults and minority communities tend to be more highly represented on Twitter than the general population [55], although minority communities have been significantly impacted by COVID-19 [56,57]. Our results should therefore be interpreted as representing a small subset of people with arthritis, and not all people with the disease. Data were extracted rather than collected directly from people with arthritis and critics of social media research purport that posts or tweets are often curated and may not be reflective of reality. We have attempted to minimize this potential bias by conducting sentiment analysis, which helped us determine the emotional tone associated with Twitter content. Regardless, sentiment analysis has its limitations: populations and individuals are constantly stimulated by their political and socioeconomic surroundings and individual demographics, which can influence the content and sentiment of people’s tweets [58,59]. We also recognize the potential limitations of our search strategy (eg, we did not search for hashtags related to specific symptoms, such as pain or function, that are not unique to arthritis) that may have impacted the number of retrieved tweets. While we had a modest sample size of tweets due to our focused study aims, sentiment analysis has previously been conducted in studies with comparable sample sizes of tweets (n=260 and n=200) [60,61]. We were unable to determine the specific diagnostic category for over half the people tweeting; while a small number of tweets were from people with osteoarthritis, it is possible that more may be represented within the “diagnosis not specified” category. We were only able to analyze tweets in English, and these largely came from high-income, developed countries. Tweets in other languages and those from people in low-and-middle income countries may provide further insights, especially where the prevalence of COVID-19 infection is high [62].

### Conclusion

This study highlights the spectrum of concerns facing people with arthritis during the COVID-19 pandemic. By exploring the content and sentiment of recent tweets, we found that individuals with arthritis conditions experience marked anxiety about medication shortages and increased physical symptom burden, and are seeking connection with and information from peers. These findings can be used to raise awareness of key issues relevant to people with arthritis during the pandemic, and to guide clinicians to tailor care that addresses the specific concerns and needs of their patients during the pandemic.

## Appendix

Multimedia Appendix 1 Twitter search strategy and results.

## Abbreviations

mHealth: mobile health
NHS: National Health Service
