# Supplementary material for: Tweets by People With Arthritis During the COVID-19 Pandemic: Content and Sentiment Analysis
Source: J Med Internet Res. 2020 Dec 3;22(12):e24550. doi: 10.2196/24550 (PMC7746504; doi:10.2196/24550)
Supplement: Multimedia Appendix 1 [file jmir_v22i12e24550_app1.docx]

**Supplementary File 1: Twitter Search Strategy and Results.**

| **Search Terms** | **Total Terms Retrieved (n)** | **Terms Excluded (n)** | **Reasons for Exclusion (n)** | **Terms Included (n=149)** |
| --- | --- | --- | --- | --- |
| #Arthritis AND #coronavirus | 127 | 74 | Tweets by organizations: 28  Tweets by individuals without arthritis: 18  Drug advertisements: 13  Tweets by clinicians: 6  Duplicate tweets: 6  Not English: 2  Tweets advertising other research: 1 | 53 |
| #Arthritis AND #COVID19 | 217 | 155 | Tweets by organizations: 73  Duplicates: 23  Tweets by individuals without arthritis: 18  Tweets by clinicians: 15  Drug advertisements: 13  Tweets advertising other research: 11  Tweets for animals with arthritis: 1  Not English: 1 | 62 |
| #Spoonie AND #coronavirus | 114 | 104 | Tweets by individuals without arthritis: 70  Tweets by organization: 24  Duplicate tweets: 8  Tweets advertising other research: 1  Not English: 1 | 10 |
| #Rheumatologist AND #Coronavirus | 14 | 6 | Tweets by organizations: 4  Tweets by clinicians: 2 | 8 |
| #Arthritis AND #Isolation | 9 | 2 | Tweets by individuals without arthritis: 2 | 7 |
| #Arthritis AND #SocialDistancing | 5 | 0 | - | 5 |
| ***Other Trialled Search Terms*** |  |  |  |  |
| #Arthritis AND #SARS-CoV-2 | 0 | 0 | - | 0 |
| #Arthritis AND #coronavirus AND #Physical Distancing | 0 | 0 | - | 0 |
| #Rheumatology AND #Coronavirus | 80 | 76 | Tweets by organization: 23  Tweets by clinicians: 23  Tweets by individuals without arthritis: 13  Tweets advertising other research: 11  Duplicates: 3  Not English: 2  Drug advertisements: 1 | 4 |
